# Effect of fresh *Triticum aestivum* grass juice on lipid profile of normal rats

**DOI:** 10.4103/0253-7613.44157

**Published:** 2008-10

**Authors:** Saroj Kothari, Anand K. Jain, Swaroop C. Mehta, Shrinivas D. Tonpay

**Affiliations:** Department of Pharmacology, Gajara Raja Medical College, Gwalior - 474 001, India

**Keywords:** Grass, hypolipidemia, wheat

## Abstract

**Objective:**

To study the hypolipidemic activity of fresh grass juice of *Triticum aestivum* in normal rats.

**Materials and Methods:**

Freshly prepared *Triticum aestivum* grass juice was administered to normal rats at the dose of 5 ml/kg and 10 ml/kg orally once daily for 21 days. Blood samples were collected after 24 hours of last administration and used for estimation of lipid profile. Fresh grass juice was also subjected to preliminary phytochemical screening.

**Results:**

Fresh grass juice administration produced dose related significant (*P* < 0.05) reduction in total chloesterol, triglycerides, low density lipoprotein-cholesterol and very low density lipoprotein-cholesterol levels in normal rats as compared to control. Preliminary phytochemical screening revealed presence of alkaloids, tannins, saponins and sterols in *Triticum aestivum* grass.

**Conclusion:**

The results of the present study lndicate hypolipidemic activity of fresh *Triticum aestivum* grass juice.

## Introduction

Fresh juice of grass (GJ) from *Triticum aestivum* Linn (Poaceae) or common wheat seed is used as a health improving adjuvant in several diseases including coronary artery disease in India as folk medicine. Hyperlipidemia is a major risk factor for coronary artery disease and is leading cause of death. Major component of total cholesterol is LDL-cholesterol, which leads to atherosclerosis.[1] Dietary wheat bran has been known to modulate hypolipidemic effect of fish oils in rats.[2] Present work was aimed to conduct preliminary phytochemical analysis and to study hypolipidemic activity of oral fresh *T. aestivum* GJ in normal rats.

## Materials and Methods

### Growing of the grass and preparation of grass juice

The grass of *T. aestivum* used in this study was grown indoors until required for experiments. Earthen pot of 12″x12″ and about 3″ depth was filled with 2½″ of growing medium composed of 3 parts of soil and one part of compost. Overnight soaked *T. aestivum* seeds were then evenly spread over it and further covered with ½″ soil. Small quantities of water were sprinkled evenly over soil and 3-4 hours indirect sunlight was allowed daily for growth of grass. On the tenth day, when grass is about 6″ tall, it is cut ½″ above the surface of soil. To harvest continuous supply of fresh grass, pots were similarly planted at one-day interval. Twenty grams of above harvested fresh grass was grounded in a laboratory mortar and the juice was squeezed out through four layers of wet muslin cloth. The residue was twice resuspended in 3 ml of sterile water and similarly squeezed. The filtrate was made to 20 ml (w/v) final volume with sterile water and administered as GJ. Each day the fresh juice was prepared prior to administration.

Fresh *T. aestivum* grass was subjected to qualitative tests by standard methods as described by Harborne[3] for preliminary phytochemical analysis

### Animals and treatment

Randomly bred six to eight weeks old Wistar rats of both sexes, weighing 140-200g, raised in the animal house of the Department of Pharmacology, Gajara Raja Medical College, Gwalior, were used for the study. These were maintained at 24±2°C with 12 h light and dark cycle and kept on standard pellet diet (Pranav Agro Industries, Delhi, India) and water *ad libitum*. The care and maintenance of animals was as per the approved guidelines of the Committee for the purpose of Control and Supervision of Experiments on Animals in India. The Institutional Animal Ethics Committee approved the protocol.

Rats were divided into three groups of six rats each and received following treatments for 21 days by gavage. The first group served as control (CG) that received normal saline, second group (T5) was given GJ at a dose of 5 ml/kg body weight and the third group (T 10) animals were given same juice at the dose of 10 ml/kg body weight. Selection of dose was based on the basis of use of fresh GJ by adult patients for various ailments as folk medicine.

### Lipid profile measurements

After 24 hours of the last dose administration the animals were anaesthetized with diethyl ether and blood samples were collected by orbital puncture. These were allowed to clot and then centrifuged at 3000 rpm/10 min, and serum was used for estimation of total cholesterol (TC) by (CHOD Pap method), triglycerides (TG) by (GPO Pap method) and high-density lipoprotein (HDL) by (Direct method) using enzymatic kits. All samples were analyzed with a spectrophotometer model BTR-830 (Biotech Spain).The concentration of low-density lipoprotein (LDL) and very low-density lipoprotein (VLDL) was calculated as described by the formula of Friedewald.[4] The baseline lipid profile was always determined prior to the treatment regime.

### Statistical analysis

Statistical evaluation was done using one-way ANOVA followed by Student-Newmen-Keul's multiple comparison tests. Differences with *P* < 0.05 were considered significant. Data are presented as mean ± SD. All statistical analysis was performed by Sigma Stat software version 2.0, Jandel Scientific Inc. USA.

## Results

Treatment of rats with GJ significantly lowered lipid parameters in T5 and T10 groups in dose related manner [Table 1]. In comparison with CG TC, TG, LDL and VLDL-cholesterol were lowered by 24, 12, 38, and 13 percent in T5 and by 48, 32, 73 and 32 percent in T10 groups respectively. Further, it was observed that HDL-cholesterol was increased by 4 in T5 and by 10 percent in T10 groups as compared to control group.

Preliminary phytochemical screening of *T. aestivum* GJ indicated the presence of alkaloids, tannins, saponins, and sterols.

**Table 1:** Effect of fresh juice of *Triticum aestivum* grass on serum lipid profile of normal rats

*Group*		*TC*	*TG*	*HDL-C*	*LDL-C*	*VLDL-C*
CG		70.18±6.44	61.20±4.94	15.25±0.78	42.69±5.97	12.24±0.98
T5		53.11±5.59^a^	53.82±4.62^a^	15.83±0.88	26.52±5.02^a^	10.76±0.92^a^
T10		36.68±4.35^a^^b^	41.77±4.91^a^^b^	16.66±0.78	11.67±4.55^a^^b^	8.35±0.98^a^^b^
One–	F	54.97	24.72	4.46	52.99	24.73
way	df	2,15	2,15	2,15	2,15	2,15
ANOVA	P	<0.001	<0.001	N.S.	<0.001	<0.001

Values are mean±S.D., n=6 in each group. The lipid profiles are given in mg/dl. *P* <0.05 (Student-Newmen-Keul's multiple comparison test)

a significant from control group

b significant fromT5 group. N.S. = not significant

## Discussion

Results clearly show that oral fresh GJ of *Triticum aestivum* has hypolipidemic properties. Though the physiological mechanism of this activity cannot be concluded from the present study and need further investigations, yet the positive presence of phytochemicals such as saponins, tannins, and sterols in GJ seem to cause these changes. Such changes are already recorded for these compounds.[5–7] Present study seems to be the first of its kind in investigating the hypolipidemic activity vis a vis preliminary phytochemical screening of *Triticum aestivum* fresh seedling grass. Further elucidation of bioactive compounds and their pharmacological activity assessment should constitute an interesting future objective.
